# Sequence capture using AFLP‐generated baits: A cost‐effective method for high‐throughput phylogenetic and phylogeographic analysis

**DOI:** 10.1002/ece3.5176

**Published:** 2019-04-16

**Authors:** Jia‐Xuan Li, Zhao‐Chi Zeng, Ying‐Yong Wang, Dan Liang, Peng Zhang

**Affiliations:** ^1^ State Key Laboratory of Biocontrol, College of Ecology and Evolution, School of Life Sciences Sun Yat‐Sen University Guangzhou China

**Keywords:** bait, *Odorrana*, phylogenetics, population genetics, sequence capture

## Abstract

Target sequence capture is an efficient technique to enrich specific genomic regions for high‐throughput sequencing in ecological and evolutionary studies. In recent years, many sequence capture approaches have been proposed, but most of them rely on commercial synthetic baits which make the experiment expensive. Here, we present a novel sequence capture approach called AFLP‐based genome sequence capture (AFLP Capture). This method uses the AFLP (amplified fragment length polymorphism) technique to generate homemade capture baits without the need for prior genome information, thus is applicable to any organisms. In this approach, biotinylated AFLP fragments representing a random fraction of the genome are used as baits to capture the homologous fragments from genomic shotgun sequencing libraries. In a trial study, by using AFLP Capture, we successfully obtained 511 orthologous loci (>700,000 bp in total length) from 11 *Odorrana* species and more than 100,000 single nucleotide polymorphisms (SNPs) in four analyzed individuals of an *Odorrana* species. This result shows that our method can be used to address questions of various evolutionary depths (from interspecies level to intraspecies level). We also discuss the flexibility in bait preparation and how the sequencing data are analyzed. In summary, AFLP Capture is a rapid and flexible tool and can significantly reduce the experimental cost for phylogenetic studies that require analyzing genome‐scale data (hundreds or thousands of loci).

## 1 INTRODUCTION

Sequencing a large number of orthologous loci across many species/individuals has become an increasingly common task in phylogenetic and ecological studies. Target sequence capture is one efficient method to fulfill this goal (Grover, Salmon, & Wendel, 2012; Jones & Good, 2016). Sequence capture hybridizes DNA or RNA baits (also called probes) to target DNA regions, physically pulls down the targeted DNA regions of interest and washes away unwanted DNA fragments so that the targeted DNA can be sequenced by high‐throughput sequencing (HTS) (Glenn & Faircloth, 2016; Lemmon & Lemmon, 2013; Mamanova et al., 2010; McCormack, Hird, Zellmer, Carstens, & Brumfield, 2013). Currently, the most popular sequence capture approaches include anchored hybrid enrichment (AHE: Lemmon, Emme, & Lemmon, 2012), ultraconserved element sequencing (UCE: Faircloth et al., 2012), and exon capture (Albert et al., 2007; Bi et al., 2012; Li, Hofreiter, Straube, Corrigan, & Naylor, 2013; Ng et al., 2009). These sequence capture approaches all use custom‐designed baits to capture a prespecified set of genomic regions that are highly conserved across diverse taxa and have been widely used in many phylogenetic studies (Bragg, Potter, Bi, & Moritz, 2016; George et al., 2011; Hedtke, Morgan, Cannatella, & Hillis, 2013; Ilves & López‐Fernández, 2014).

However, the custom‐bait‐based methods require reference genomic sequence of the target regions for bait design and the cost of bait synthesis is normally high (Jones & Good, 2016; Lemmon & Lemmon, 2013; McCormack et al., 2013). To skip the bait design and reduce experiment cost, some researchers have attempted to use homemade baits to implement target capture experiments, for instance, using PCR products as baits (Amplicon capture: Mariac et al., 2014; Maricic, Whitten, & Pääbo, 2010; Peñalba et al., 2014; Tsangaras et al., 2014), using restriction site‐associated DNA fragments (RAD) as baits (hyRAD: Suchan et al., 2016), or using cDNA fragments as baits (Puritz & Lotterhos, 2018). These studies show that using homemade baits for sequence capture is feasible. Therefore, developing more methods to simply and reliably generate homemade baits for sequence capture is of importance to the ecology and evolution research community.

The AFLP (amplified fragment length polymorphism) technique was established as a highly efficient genomic fingerprinting method in 1995 (Vos et al., 1995). Because the AFLP method is suited for fingerprinting genomic DNA of any origin and complexity, it has been widely used for applications in genetic analysis such as addressing genetic relationship, displaying population structure, and assessing genetic diversity (Mueller & Wolfenbarger, 1999; Vuylsteke, Peleman, & van Eijk, 2007; Zhang, van Parijs, & Xiao, 2014). The AFLP technology is based on selective PCR amplification of restriction fragments from the digested genomic DNA. One major advantage of this fingerprinting method is that no prior genomic information is needed and the number of amplified fragments can be controlled by the choice of restriction enzymes and the number of selective bases used in the amplification process (Vos et al., 1995). Methodologically, AFLP is essentially a genome‐reduced representation method combined with PCR and thus can potentially be used to generate baits for sequence capture.

In this study, we develop a novel approach called “AFLP‐based genome sequence capture (AFLP Capture),” in which AFLP fragments are used as baits for sequence capture. This method does not require any prior genome information and can generate controllable number of fragments as capture baits by selective PCR amplification. We also propose a bioinformatic pipeline based on mutual‐BLAST for converting the AFLP Capture data into orthologous sequences or SNPs. We test the utility of this method by displaying individual genetic difference and resolving species relationship in the genus *Odorrana* (Amphibia, Anura, Ranidae). Our results demonstrate that AFLP Capture is capable of producing hundreds of orthologous loci or tens of thousands of SNPs for genetic analysis at both interspecies and intraspecies levels. The method presented here provides a rapid, scalable, and cost‐effective way for high‐throughput genetic analysis in nonmodel organisms.

## 2 MATERIALS AND METHODS

### 2.1 Samples and DNA extraction

Here, we wanted to test the experimental performance of the AFLP Capture method at two different phylogenetic levels: interspecies and intraspecies. For the interspecies level, we used eleven different *Odorrana* (Amphibia, Anura, Ranidae) species: *O. huanggangensis, O. schmackeri, O. hainanensis, O. anlungensis, O. margaretae, O. yizhangensis, O. grahami, O. chloronota, O. graminea, O. versabilis,* and *O. tormota*. This taxon sampling represents the evolutionary divergence within a genus. Two distantly related species from the same family, *Babina pleuraden* and *Rana altaica,* were used as outgroup. For the intraspecies level, we selected four individuals of *O. huanggangensis* (including the previous *O. huanggangensis* sample) collected from different localities in China. So, there are a total number of 16 *Odorrana* samples used in this study (Table 1).

**Table 1:** Taxon sampling, sequencing results, and detailed information from the bioinformatic pipeline for each sample

Species	Voucher	Locality	Raw reads	Filtered reads	% Duplicates	Number of contigs with length >200 bp read coverage >5×	Number of contigs passed MBH	Average target contig length	% of reads on‐target
*O. huanggangensis* a	SYS a005999	Daiyunshan, Fujian	8,691,898	8,013,128	6.3	5,030	–	1,197	73.17
*O. huanggangensis*	SYS a002865	Yongzhou, Hunan	9,436,975	8,585,416	7.5	6,832	1,880	1,455	72.78
*O. huanggangensis*	SYS a004335	Tongren, Guizhou	7,217,890	6,686,734	5.8	3,704	1,341	1561	66.41
*O. huanggangensis*	SYS a001689	Tongboshan, Jiangxi	7,673,298	7,041,130	6.7	4,994	1679	1,477	67.69
*O. schmackeri*	SYS a005476	Yichang, Hubei	9,684,879	8,765,254	8.0	6,192	1602	1582	70.51
*O. hainanensis*	SYS a002260	Diaoluoshan, Hainan	5,492,800	5,108,580	5.5	2,801	991	1579	65.32
*O. anlungensis*	SYS a005177	Baise, Guangxi	10,267,913	9,422,046	6.7	6,065	1,213	1603	63.25
*O. margaretae*	SYS a004291	Zhangjiajie, Hunan	5,921,789	5,551,510	4.7	3,122	941	4,211	70.42
*O. yizhangensis*	SYS a003171	Jinggangshan, Jiangxi	9,786,804	8,634,074	10.3	1,577	795	1,742	71.93
*O. grahami*	SYS a005827	Anyuan, Yunnan	7,010,871	6,249,492	9.4	1541	673	1,673	65.18
*O. chloronota*	SYS a003881	Yingjiang, Yunnan	6,015,579	5,524,640	6.7	1,261	548	1,587	63.07
*O. graminea*	SYS a005271	Wuzhishan, Hainan	10,313,408	9,347,380	7.9	899	454	1,374	60.04
*O. versabilis*	SYS a005118	Guilin, Guangxi	11,193,412	10,257,230	6.9	1,246	569	1,918	57.26
*O. tormota*	SYS a002700	Huangshan, Anhui	11,184,169	10,257,392	6.8	1,453	579	1,951	54.46
*B. pleuraden*	SYS a003775	Gaoligongshan, Yunnan	10,626,830	9,613,774	8.1	1,349	275	2,084	42.08
*R. altaica*	AM33	Kanas, Xinjiang	9,734,670	8,679,812	9.4	712	172	2,474	33.82

a Sample that was used to generate the AFLP baits in this study.

Total genomic DNA was extracted from ethanol‐preserved liver or muscle tissue, using the TIANamp Genomic DNA Kit (TIANGEN Inc., Beijing, China). All DNA extracts were diluted to a concentration of 10–50 ng/μl with 1× TE and stored at −20°C for further use.

### 2.2 AFLP bait preparation

**Figure 1:**
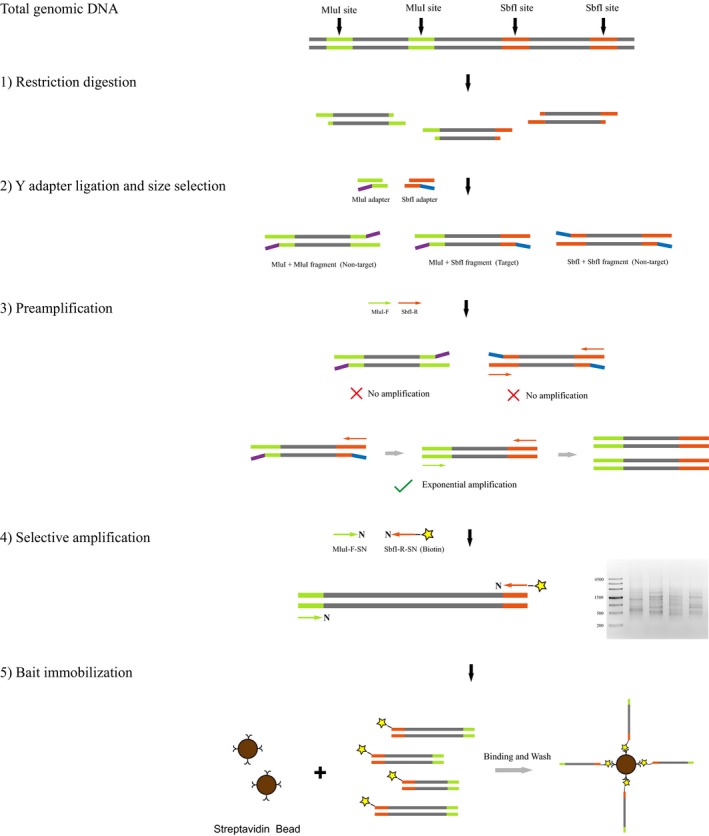
Schematic overview of the AFLP bait preparation. Detailed protocol is given in Section 2

The workflow of producing AFLP baits is illustrated in Figure 1. Because the quality of the genomic DNA is crucial for the outcome of AFLP experiment, we extracted high molecular weight DNA (fragments ~20 KB) from ethanol‐preserved tissue of an *O. huanggangensis* individual (Fujian) for AFLP bait preparation. Genomic DNA (1 μg) was digested for 20 min at 37°C with 1 μl of FastDigest MluI (Thermo Fisher Scientific), 1 μl of FastDigest SbfI (Thermo Fisher Scientific), and 2 μl 10× FastDigest buffer (Thermo Fisher Scientific) in a total volume of 20 μl. The digestion was purified using AMPure XP beads (Beckman Coulter), with a ratio of 1.8:1 to the sample, followed by resuspension in 50 μl of ddH_2_O. Then, restriction‐associated double‐stranded Y adapters were ligated to the purified fragments in a 60 μl reaction containing 10 μM MluI adapter, 10 μM SbfI adapter, 5U T4 DNA ligase (Fermentas Inc.), 100 mM ATP, and 1× T4 ligation buffer. Ligation reactions were incubated for 30 min at 25°C. The 60 μl ligation reaction was subsequently size‐selected for 500‐ to 2,000‐bp fragments with AMPure XP beads by separately adding 15 μl of beads (discard beads) and 20 μl of beads (keep beads) to the solutions. Finally, the size‐selected products were eluted from the air‐dry beads with 50 μl of 1× TE and used as templates in the next step.

In the preamplification step, the design of Y adapters ensures that only those fragments ligated with both SbfI and MluI adapters at the two ends can be successfully amplified (Figure 1). The PCR reaction mixture consisted of 1.25 U HiFi Taq DNA Polymerase (TransGen Inc.), 1× HiFi PCR buffer, 0.2 mM dNTPs, 0.2 μM MluI‐F primer, 0.2 μM SbfI‐R primer, and 5 ng template DNA in a total volume of 25 μl. The thermal cycling program included the following steps: an initial denaturation for 4 min at 94°C followed by 20 cycles of 30 s at 94°C, 1 min at 56°C, 1 min at 72°C, and a final extension of 10 min at 72°C. After this preamplification step, the reaction mixtures were diluted 10‐fold with 10 mM Tris‐HCl, 0.1 mM EDTA pH 8.0, and used as templates for the selective amplification.

Two forward (MluI‐F‐SA and MluI‐F‐ST) and two reverse (SbfI‐R‐SC and SbfI‐R‐SG) selective primers (each has a selective nucleotide at the 3′ end) were used in the selective amplification. So there were four primer combinations. To obtain single‐stranded baits in the next step, only the reverse selective primers were labeled with 5′ biotin. The reaction mixture for selective amplification contained 1 x HiFi PCR buffer, 0.2 mM dNTPs, 1.25 U HiFi Taq DNA Polymerase, 0.2 μM of forward selective primer, 0.2 μM reverse selective primer, and 2 μl of 10‐fold diluted preamplification product in a total volume of 25 μl. The thermal cycling program included the following steps: an initial denaturation for 4 min at 94°C followed by 36 cycles of 30 s at 94°C, 30‐s annealing, 1 min at 72°C, and a final extension of 10 min at 72°C. The annealing temperature in the first cycle was 65°C, then subsequently reduced in each cycle by 0.7°C for the next 12 cycles, and set at 56°C for the remaining 23 cycles. The selective amplification products were purified by AMpure XP beads and checked on a 1.2% TAE agarose gel. After that, four different selective amplification products (AFLP fragments) were pooled together in equal concentrations based on quantification with a NanoDrop 2000 spectrophotometer.

In the step of bait immobilization, 40 μl of biotinylated AFLP fragments (2 μg) was mixed with 40 μl of 2× BWT buffer (2 M NaCl, 10 mM Tris‐Cl, 1 mM EDTA, 0.1% Tween 20). The double‐stranded AFLP fragments were denatured by 5‐min incubation at 95°C and quickly transferred to ice bath. 50 μl of Dynabeads MyOne streptavidin magnetic beads (Life Technologies) was washed with 800 μl 1× BWT buffer and washed again with 800 μl TET buffer (10 mM Tris‐Cl, 1 mM EDTA, 0.05% Tween 20). The denatured AFLP fragments were then mixed with the washed MyOne beads and incubated for 20 min at 25°C to allow the biotinylated AFLP baits to bind to the beads. The beads were then washed four times with PWB buffer (0.1 M NaCl, 5 mM Tris‐Cl, 0.5 mM EDTA, 0.05% Tween 20) at 65°C to remove the nonbiotinylated strands. Finally, the bait‐coated beads were resuspended in 50 μl TET buffer and stored at 4°C.

### 2.3 Library preparation, hybridization capture, and sequencing

Unlike the AFLP bait preparation which requires high‐quality DNA, sequencing library preparation can use degraded DNA samples. For each sample to be captured, genomic DNA (100 ng) was randomly fragmented to 200–400 bp with 1 μl NEBNext dsDNA Fragmentase (NEB) at 37°C for 14 min in a 20 μl reaction volume. The reaction was stopped by adding 2 μl 0.5 M EDTA and then purified by AMpure XP beads. Purified DNA was used in NEBNext Ultra DNA Library Prep Kit for Illumina library preparation with no modifications.

For each sample, 500 ng of library and 5 μl of bait‐immobilized beads were used for sequence capture. Hybridization capture conditions are based on previously published protocols (Li et al., 2013; Maricic et al., 2010; Peñalba et al., 2014) with some modifications. In our experiment, each sample was enriched only once and the hybridization reaction started at 65°C and was decreased by 3°C every 6 hr over 36 hr and ended at 50°C. This program was implemented to gradually decrease hybridization stringency to increase the likelihood of capturing fragments with higher sequence divergence from the baits. After the enrichment, each sample was amplified with indexed primers so that all samples could be pooled together for sequencing. Detailed protocol for bait preparation and hybridization capture is available at https://figshare.com/s/5a4eee383e2dc9afba53. All final enriched and indexed libraries were pooled in equimolar concentrations. The pooled libraries were later sequenced on an Illumina HiSeq X‐ten lane using paired‐end 150‐bp mode, with other libraries that are not part of this study.

### 2.4 Bioinformatic workflow

#### 2.4.1 Reads assembly

Sequence reads were demultiplexed by the 8‐bp index that was incorporated with each sample during PCR amplification. Adapter sequences, low‐quality nucleotides, and PCR duplicates of the raw reads were removed by using Trimmomatic (Bolger, Lohse, & Usadel, 2014), FastQC (http://www.bioinformatics.babraham.ac.uk/projects/fastqc/), and FastUniq (Xu et al., 2012). The filtered reads for each sample were assembled into contigs using the SPAdes version 3.8.1 genome assembler (Bankevich et al., 2012), which automatically selects a K‐mer value based on read length and data type (‐‐cov‐cutoff auto). The obtained contigs were further filtered with CD‐HIT‐EST (Fu, Niu, Zhu, Wu, & Li, 2012) to reduce redundancy (95% similarity cutoff). The sequencing depths for the filtered contigs were calculated by SAMtools version 1.4.1 (Li et al., 2009). Only contigs of length ≥200 bp and average sequencing depth ≥5× were retained for further analysis.

#### 2.4.2 Identification of orthologous sequences (for interspecies level)

**Figure 2:**
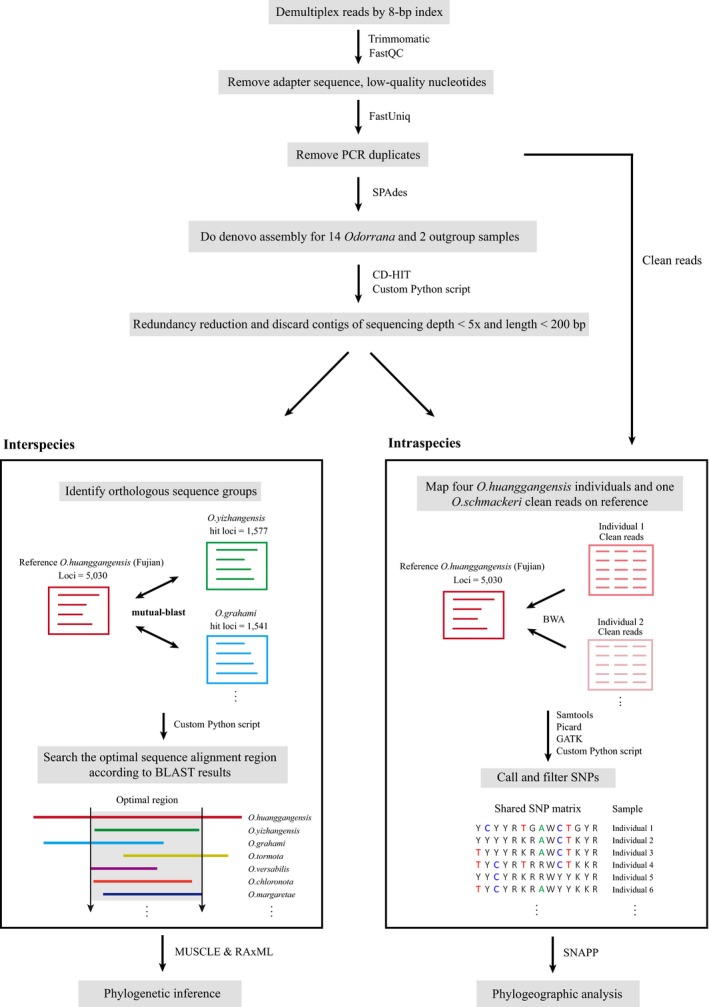
Bioinformatic pipeline used to process the AFLP Capture data. For interspecies level analysis, the data were eventually converted into orthologous sequence groups (OGs). For intraspecies level analysis, the clean reads of different individuals were mapped onto the reference sequences to call SNPs. Detailed protocol is given in Materials and methods

In a previous study, Suchan et al. (2016) used RAD fragments as bait for sequence capture and they showed that sequencing the bait is optional. According to this suggestion, on interspecific level, our experimental design does not use the AFLP bait sequences as reference for read mapping, but use bidirectional BLAST to identify orthologous sequences (see discussion). We regarded the filtered contigs captured from the bait species *O. huanggangensis* (Fujian) (a total number of 5,030 contigs; Figure 2) as reference and used a mutual best‐hit (MBH) strategy (program = BLASTn, expectation value = 1E−10; NCBI BLAST+ version 2.6.0, Boratyn et al., 2013) to identify 1:1 orthology groups (OGs) from all samples. We defined orthology as being likely if two contigs from a sampled species and the reference species *O. huanggangensis* (Fujian) find each other as the best hit in the bidirectional BLAST.

#### 2.4.3 Search for optimal aligning region for each OG

Within each OG, the contig sequences are normally different in length, which makes them difficult to align. We wrote a Python script (available at https://figshare.com/s/5a4eee383e2dc9afba53) to determine the optimal aligning region for all sequences within an OG. In brief, the script uses the mutual‐BLAST results to determine the relative position of each sequence to the reference sequence and searches for the optimal upstream and downstream boundaries to trim the sequences (Figure 2). The trimming condition of the upstream and downstream boundaries is adjustable. Here, we demand that at least 50% of species have data at both the upstream and downstream boundaries.

#### 2.4.4 Phylogenetic analysis

For each OG, trimmed sequences were aligned using MUSCLE version 3.8 (Edgar, 2004) with default settings. Some OG alignments may still contain problematic sequences because of wrong orthology assignment or assembling errors. To circumvent this problem, a sequence was discarded if its average similarity to all other sequences within the alignment is below 30% (this cutoff value is somewhat arbitrary, but we think it is reasonable to remove sequences of such a low similarity). After realigning the retained sequences, we kept only the alignments that contain more than six taxa.

The refined alignments were concatenated into a final supermatrix. Phylogenetic trees were constructed using the maximum‐likelihood (ML) method under the GTRGAMMA nucleotide substitution model in RAxML version 8.0 (Stamatakis, 2014). Branch support for the resulting phylogeny was evaluated with 500 rapid bootstrapping replicates (‐f a option) implemented in RAxML.

#### 2.4.5 SNP calling (for intraspecies level) and Phylogeographic analysis

The filtered contigs captured from the *O. huanggangensis* (Fujian) sample that was used for bait preparation (a total number of 5,030 contigs; Figure 2) were used as reference for SNP calling. The clean reads of the four *O. huanggangensis* individuals and one *O. schmackeri* were mapped to the reference using BWA version 0.7.5a (Li & Durbin, 2009) with default parameters. Read sort, BAM file conversion, and read duplicate mask were done by using the SAMtools and the Picard toolkit (http://broadinstitute.github.io/picard/). Base quality score recalibration, local realignment, and SNP calling using standard hard filtering parameters were carried using Genome Analysis Toolkit 3 (GATK‐3.2.2: McKenna et al., 2010). GATK Best Practices recommendations were followed (Van der Auwera et al., 2013; Depristo et al., 2011). An open source Python script (vcf2phylip.py; available at https://github.com/edgardomortiz/vcf2phylip) was used to convert the VCF file outputted by GATK to a shared SNP matrix in nexus format. The relationships among different *O. huanggangensis* individuals were inferred using the SNAPP template (Bryant, Bouckaert, Felsenstein, Rosenberg, & Roychoudhury, 2012) implemented in BEAST 2.1.3 (Bouckaert et al., 2014). The SNAPP analysis was run for 1,000,000 iterations. The first half of the trees was discarded as burn‐in.

## 3 RESULTS

We obtained a total of 140 million paired‐end 150‐bp reads (~21 Gb of sequence data) for the hybridization capture libraries, successfully demultiplexing 16 samples. The total base pair yield is averagely 1,314.9 Mb across the 16 samples (range: 823.9–1679.0 Mb). After filtration of the low‐quality reads, the average total base pair yield per sample ranged from 766.9 Mb to 1538.6 Mb with a mean of 1,197.5 Mb and a variation of approximately twofold.

Clean reads of each sample were assembled into contigs using the SPAdes genome assembler. After filtered by redundancy reduction, sequencing depth (>5×), and contigs length (>200 bp), the numbers of contigs ranged from 712 to 6,832 for different samples. The *O. huanggangensis* (Fujian) sample that was used for bait preparation yielded a total number of 5,030 filtered contigs. Using these 5,030 filtered contigs as reference sequences, the other three *O. huanggangensis* samples had 1,341, 1,679, and 1,880 contigs that passed MBH, respectively. The numbers of contigs that passed MBH for other ten *Odorrana* species ranged from 454 to 1,602. The two outgroup species only had 172 and 275 contigs that passed MBH, probably because they are too genetically distant to the bait species *O. huanggangensis* (Fujian). The contigs that passed MBH are regarded as the final target loci for AFLP Capture. The average length for these target sequences is 1885 bp. The average percentage of on‐target reads is 62.34%, with the lowest value of 33.82% in *R. altaica* and the highest value of 72.78% in *O. huanggangensis* (Hunan). A summary of the assembly, MBH analyses, and mapping statistics is shown in Table 1.

**Figure 3:**
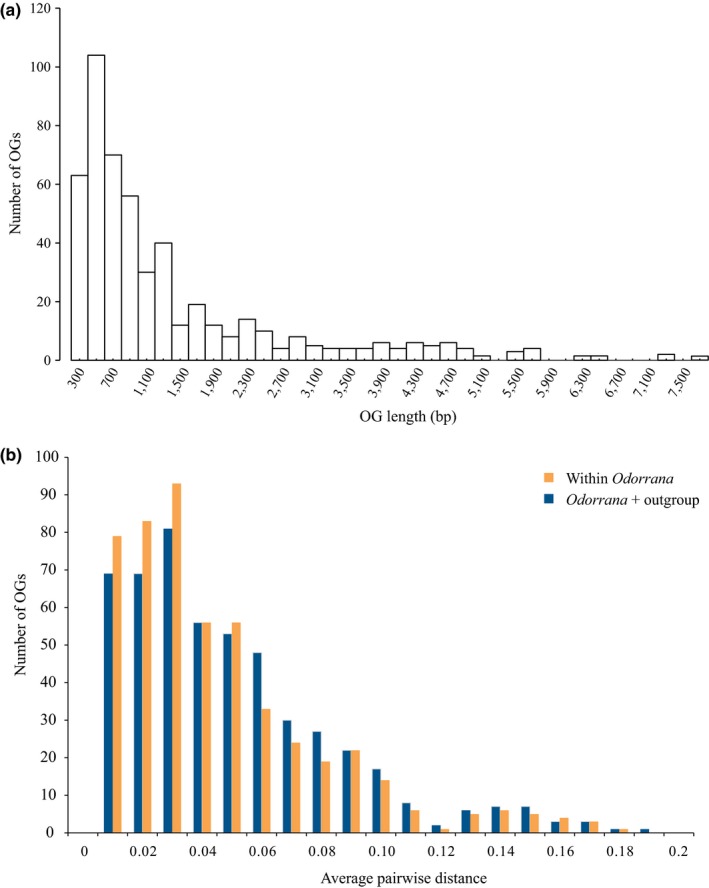
Data characteristic of the obtained 511 orthologous sequence groups (OGs). (a) Length distribution of the 511 OGs. (b) Average pairwise distance distribution of the 511 OGs within *Odorrana* (orange) and with outgroup (blue), respectively

**Figure 4:**
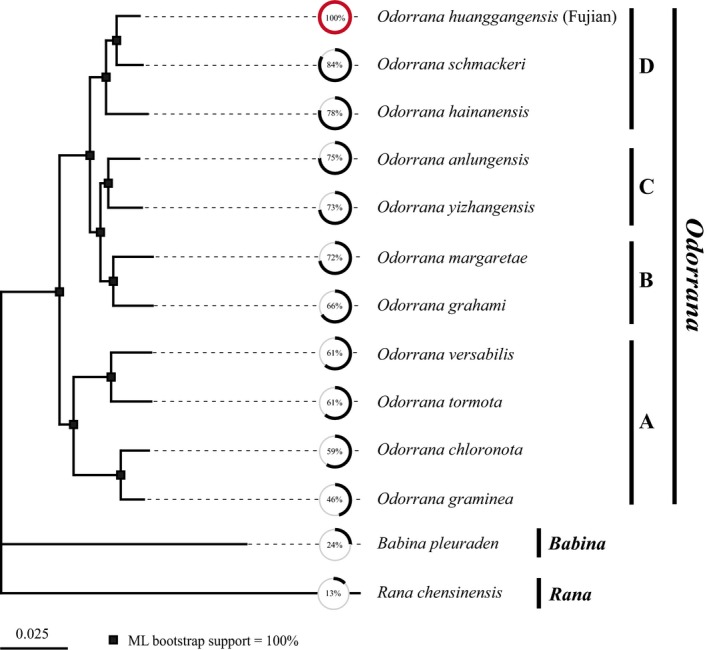
Phylogenetic tree reconstructed from ~713 kb of DNA sequence data for 11 *Odorrana* species and two outgroup species using RAxML. The circles on the dashed represent the completeness of data (percentage of the obtained loci to the 511 target OGs) for each species. The bait species *O. huanggangensis* (Fujian) is indicated with a red circle

We first investigated the effectiveness of AFLP Capture to generate multilocus data set for phylogenomic analysis across a moderate evolutionary divergence. From the obtained target contigs, 511 OGs were identified that included at least six species of the 11 *Odorrana* species sampled in this study. The lengths of the 511 OGs ranged from 203 to 7,779 bp, with an average of 1,378 bp (Figure 3a). Approximately 20% of the OGs were more than 2,000 bp, longer than the bait fragments (500–2,000 bp), which suggests that some captured loci contained flanking sequences of their bait regions. The evolutionary rates of these 511 OGs, measured by overall mean p‐distance, ranged from 0.0001 to 0.1709 within the genus *Odorrana* and ranged from 0.0002 to 0.1883 when outgroup was included (Figure 3b). As a reference gene, the mitochondrial COI has an evolutionary rate of 0.169 within *Odorrana*, which indicates that most of our captured nuclear loci evolve slower than COI. The concatenated supermatrix of the 511 OGs was 713,111 bp in length, 71% complete by locus and species or 58% complete by characters. ML analysis produced a well‐resolved phylogeny for the 11 *Odorrana* species. All the 10 internal nodes were strongly supported with bootstrap values of 100% (Figure 4). We recognized four major clades within *Odorrana* (A‐D; Figure 4), which was repeatedly found in previous *Odorrana* studies based on mtDNA data (Cai, Che, Pang, Zhao, & Zhang, 2007; Chen et al., 2013; Jiang & Zhou, 2005; Matsui, Shimada, Ota, & Tanaka‐Ueno, 2005), nuclear data (Stuart, 2008), and combinations of mtDNA and nuclear data (Che et al., 2007; He, 2017; Pyron & Wiens, 2011; Wiens, Sukumaran, Pyron, & Brown, 2009). The relationships among the four clades is (A,((B,C),D)), most resembles the result of (A,(B,(C,D))) inferred from 14 nuclear genes (He, 2017).

**Figure 5:**
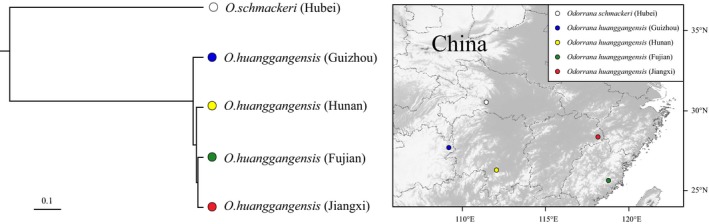
Collection localities and interrelationships of four *O. huanggangensis* individuals from Guizhou (blue), Hunan (yellow), Fujian (green), and Jiangxi (red). The tree was reconstructed from 7,351 high‐quality SNPs using SNAPP. The *O. schiackeri* was used as outgroup (white)

We then explored the capability of AFLP Capture in discovering SNPs for phylogeographic analysis at population level. Using the 5,030 filtered contigs of *O. huanggangensis* (Fujian) as reference, we identified a total of 123,290 raw SNPs from the other three *O. huanggangensis* individuals and one *O. schmackeri* individual, with 104,354 SNPs having a quality score higher than 30. Of the 104,354 high‐quality SNPs, 7,351 had zero missing data in the five samples and were used in the SNAPP analysis. Using *O. schmackeri* as outgroup, the SNAPP result suggested that the two *O. huanggangensis* individuals sampled from Fujian and Jiangxi are sister taxa and closely related to an individual sampled from Hunan. The *O. huanggangensis* individual from Guizhou is more distantly related to the other three individuals, which was in line with the geographical distance across these *O. huanggangensis* individuals (Figure 5). This preliminary result indicates that the AFLP Capture data can also provide substantial amount of SNPs for population genetic studies or phylogeographic studies among closely related species.

## 4 DISCUSSION

Our study demonstrates that anonymous AFLP fragments can be used as capture baits to efficiently enrich a wealth of orthologous loci at moderate evolutionary scale, without the need of prior genomic information. In the case of *Odorrana*, our AFLP Capture method can not only provide several hundreds of orthologous loci to resolve the phylogenetic relationships within the genus, but also generate tens of thousands of high‐quality SNPs to display genetic difference among individuals. These results suggest that AFLP Capture can be applied in both phylogenetic and population genetic studies of nonmodel organisms.

A target sequence capture experiment normally included two steps: (a) bait preparation; (b) library preparation, hybridization enrichment, and sequencing. Our study aims to provide a new solution for the first step of bait preparation but not the later step. In terms of bait preparation, most previously published methods used commercially synthesized baits, such as UCE (Faircloth et al., 2012) and AHE (Lemmon, Emme, & Lemmon, 2012). Although the cost of commercial baits can be diluted by applying them to more samples, the initial investment is high (normally ~$2,400). In contrast, to generate a set of AFLP fragments as capture baits, researchers just need to choose one bait species related to the organism group, of their interest. Following the five steps (DNA digestion, adapter ligation, size selection, preamplification, and selective PCR), the bait preparation can be done in no more than 2 days. The initial investment for the reagents (restriction enzymes, adapter, and PCR primer oligos) is low, ~$300 in our laboratory. Compared to UCE/AHE baits, using homemade AFLP bait can reduce the cost of the bait preparation step of a sequence capture experiment. However, UCE/AHE bait can work across deep to shallow divergences while AFLP bait is highly taxon‐specific and only suitable for moderate to shallow divergence. In recent years, many taxonomic and ecological studies focus on shallow‐scale questions of nonmodel organisms, which often lacks applicable UCE/AHE bait sets. Under these circumstances, our AFLP Capture method is particularly helpful because it can generate homemade capture baits without the need for prior genome information.

The key of using restriction fragments as capture baits is how to control the number of the DNA fragments. Recently, Suchan et al. (2016) attempted to use RAD fragments as baits to capture homologous fragments from genomic shotgun sequencing libraries (hyRAD). To obtain a controllable number of RAD fragments, they used the Pippin Prep electrophoresis platform (Sage Science) to recover a sharp band (peaking at 270 bp) from the RAD digestion. Considering that such a specialized equipment is unusual for most laboratories, we followed their protocol to recover a sharp RAD band (peaking at 300 bp) by manual gel cutting for several times, using the same batch of RAD mixture. After sequencing the recovered RAD fragments with Illumina HiSeq, we found that the numbers of the RAD fragments from different batches varied greatly, from 6,840 to 14,040 (our unpublished results). This result suggests that using size selection to control the number of restriction fragments is difficult to repeat without the support from specialized equipment. Under the condition of manual manipulation, different batches of RAD baits may contain quite different numbers of RAD fragments.

Our AFLP Capture method controls the number of restriction fragments mainly by selective PCR amplification. Although our method also includes a step of size selection, its purpose is just to remove small restriction fragments (length <500 bp). In our method, the key of controlling the number of DNA fragments is selective PCR amplification. Compared to the size selection strategy (using regular laboratory electrophoresis equipment), using selective PCR to control the number of baits is easier to manipulate and more repeatable (Zhang et al., 2014). In the selective PCR step of this study, each reaction contains a pair of selective primers with a single selective nucleotide at the 3’ end (e.g., MluI‐F‐SA/ SbfI‐R‐SC), which can theoretically amplify one‐sixteenth of the total digestion fragments. If one wants to decrease the complexity of the baits (fewer loci), one can increase the number of selective nucleotide to 2 or 3 to recover smaller proportion of the total fragments. On the contrary, if one needs to increase the complexity of the baits (more loci), one can pool different combinations of the selective PCR products. In this study, the PCR products of four selective primer combinations were pooled together, so we actually obtained a quarter of the total fragments. However, to ensure the capture efficiency, the number of bait fragments cannot increase without limit. According to our laboratory experience, the optimal number of bait fragments is between 500 and 5,000. And higher complexity of the baits also means that more sequencing data are needed to get sufficient coverage.

**Figure 6:**
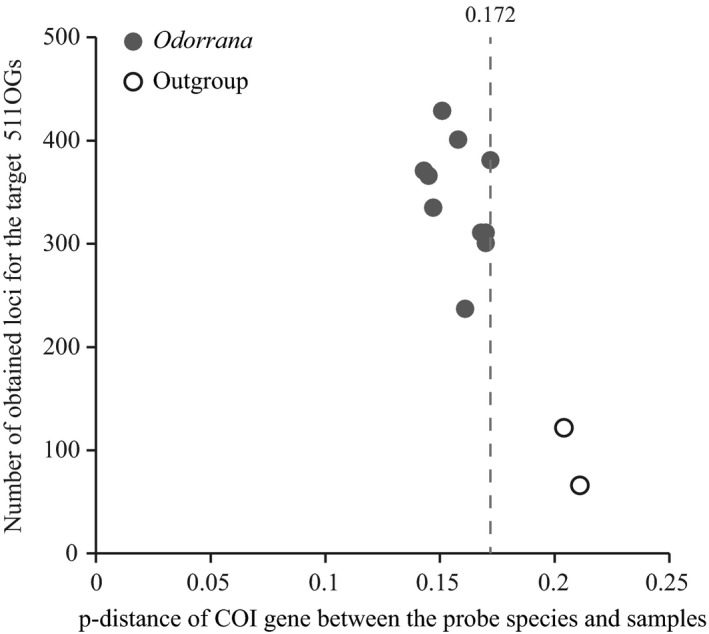
The efficiency of AFLP Capture for the *Odorrana* species and the outgroup species. Each dot represents a species. The capture efficiency for each species is measured by the number of obtained loci per sample to the 511 target OGs. It appears that the capture efficiency greatly decreases when the p‐distance of mitochondrial COI gene between the bait species *O. huangganggensis* (Fujian) and the sample is larger than 0.172

Unlike custom‐designed baits, AFLP baits are anonymous genomic loci. So it is hard to estimate sequence divergence between AFLP baits and the target DNAs. If some species are too distantly related to the species that is used for bait preparation, the anonymous AFLP baits may not work well for them. Our demonstration case is the genus *Odorrana* of which divergence is ~22 million years (He, 2017). However, genera are subjective and can be very young or very old, and moreover, different organisms have various evolutionary rates. It is not appropriate to say that AFLP Capture works well at genus level or work well for a group of less than 20 million years. We suggest sequencing a common DNA molecular marker such as mitochondrial COI to evaluate the rough sequence divergence between the probe species and other samples. In our case, when the mitochondrial COI divergence between the probe species and some of our samples (outgroup) exceeds 20%, the AFLP Capture method recovers no more than 25% of the 511 target OGs (Figure 6). However, when the COI divergence is below 17.2%, the target locus recovery rate increases to 50% ‐ 85% (Figure 6). This threshold value (20% COI divergence) can be used to predict whether the AFLP bait can effectively capture the target sequence.

Most target capture methods use the sequences of baits as reference to map the capture data (Faircloth, 2015; Faircloth et al., 2012; Johnson et al., 2016; Lemmon et al., 2012). It is worth pointing out that our AFLP Capture method does not sequence the AFLP fragments to get the reference sequences of the AFLP baits. Instead, we use a mutual best‐hit (MBH) strategy to identify 1:1 orthology groups (OGs) from all samples. One of the reasons for doing so is because AFLP baits are often noncoding sequences, thus more phylogenetically distant specimens are difficult for read mapping. Moreover, in sequence capture experiments, the capture data often contain flanking sequences of the bait regions. If the capture sequencing data are directly mapped to the bait reference sequences, the flanking sequences of the bait regions will not be kept. Alternatively, if the sequencing data are first assembled into large contigs and then assigned to orthologous groups using the MBH strategy, the flanking sequences of the bait regions can be kept and used in the subsequent analysis. In fact, before this study, we had a small pilot experiment to compare the difference in the utilization rate of the AFLP Capture data using the two different strategies: (a) mapping data to bait reference and (b) identifying orthologous contigs by MBH. The pilot experiment included only eight samples, and the AFLP bait was sequenced. We identified 94 OGs (a total length of 89,168 bp with zero missing data) by mapping data to bait reference and 164 OGs (a total length of 216,328 bp with zero missing data) by MBH. This rough result showed that using MBH rather than direct mapping can obtain more OGs and a longer concatenated data matrix from AFLP Capture data. However, because the pilot experiment is small, further comparison between these two data processing strategies may be needed in the future.

In summary, we presented a method of using the AFLP technique to generate a large number of anonymous genomic fragments as capture baits. Compared to the other target capture methods using commercial synthesis baits, such as AHE (Lemmon et al., 2012), UCE capture (Faircloth et al., 2012), and exon capture (Albert et al., 2007; Bi et al., 2012; Li et al., 2013; Ng et al., 2009), our approach has the benefits of less expensive and more flexible bait preparation steps without the need of prior genome information. The AFLP Capture method can recover hundreds to thousands of homologous loci from relatively diverged taxa, making it particularly suitable to study shallow‐scale divergence events, such as the relationships within a genus or a species complex. We hope that this method can serve as a new tool of data collection for the evolutionary biologists working in the era of high‐throughput sequencing.

## CONFLICT OF INTEREST

None declared.

## AUTHOR CONTRIBUTIONS

P.Z., J.X.L., and D.L. designed the research. Y.Y.W. provided the tissue samples. J.X.L. and Z.C.Z. performed the experiment. J.X.L. analyzed the data. J.X.L., D.L., and P.Z. wrote the manuscript.

## DATA ACCESSIBILITY

Raw read data were deposited in NCBI SRA (accession PRJNA503087). Experiment protocol, python script, the concatenated data matrix, the SNPs matrix, raw capture contigs, and the resulting phylogenetic trees were deposited in FigShare https://figshare.com/s/5a4eee383e2dc9afba53.
